# Genome sequence of the sugarcane aphid, *Melanaphis sacchari* (Hemiptera: Aphididae)

**DOI:** 10.1093/g3journal/jkae223

**Published:** 2024-09-18

**Authors:** Jinshuai Zhao, Liqiang Xie, Xinrui Zhao, Luhua Li, Jianghui Cui, Jinfeng Chen

**Affiliations:** State Key Laboratory of Integrated Management of Pest Insects and Rodents, Institute of Zoology, Chinese Academy of Sciences, Beijing 100101, China; College of Life Sciences, University of Chinese Academy of Sciences, Beijing 100049, China; State Key Laboratory of Integrated Management of Pest Insects and Rodents, Institute of Zoology, Chinese Academy of Sciences, Beijing 100101, China; College of Life Sciences, University of Chinese Academy of Sciences, Beijing 100049, China; North China Key Laboratory for Crop Germplasm Resources of Education Ministry, Hebei Agricultural University, Baoding, Hebei 07100, China; College of Agriculture, Guizhou University, Guiyang 550025, China; North China Key Laboratory for Crop Germplasm Resources of Education Ministry, Hebei Agricultural University, Baoding, Hebei 07100, China; State Key Laboratory of Integrated Management of Pest Insects and Rodents, Institute of Zoology, Chinese Academy of Sciences, Beijing 100101, China

**Keywords:** the sugarcane aphid, *Melanaphis sacchari*, synteny, genome assembly

## Abstract

The sugarcane aphid, *Melanaphis sacchari*, is an agricultural pest that causes damage to plants in the Poaceae (the grasses) family, such as sorghum and sugarcane. In this study, we used nanopore long reads and a high-throughput chromosome conformation capture chromatin interaction maps to generate a chromosome-level assembly with a total length of 356.1 Mb, of which 85.5% (304.6 Mb) is contained within the 3 autosomes and the X chromosome. Repetitive sequences accounted for 16.29% of the chromosomes, and a total of 12,530 protein-coding genes were annotated, achieving 95.8% Benchmarking Universal Single-Copy Ortholog gene completeness. This offered a substantial improvement compared with previous low-quality genomic resources. A phylogenomic analysis by comparing *M. sacchari* with 24 published aphid genomes representing 3 aphid tribes revealed that *M. sacchari* belonged to the tribe Aphidini and maintained a conserved chromosome structure with other Aphidini species. The high-quality genomic resources reported in this study are useful for understanding the evolution of aphid genomes and studying pest management of *M. sacchari*.

## Introduction

Aphids (Hemiptera: Aphididae) are one of the most destructive agricultural pests. They feed on plant phloem (phloem-feeding) and transmit more than 55% of all known insect-transmitted plant viruses (Ng and Falk 2006; Mathers *et al*. 2020). About 100 out of the ∼5,000 aphid species have been recognized as important agricultural pests (Blackman and Eastop 2017). Additionally, the complex life cycle, extensive phenotypic plasticity, and rapid environmental adaptation of aphids make them an important model for the study of plant–insect interactions (Ferry *et al*. 2004; Hogenhout and Bos 2011), speciation (Hawthorne and Via 2001; Peccoud *et al*. 2009), and sex chromosome evolution (Jaquiery *et al*. 2013, 2018).

There is a lack of genomic resources on aphids, which hampers the study of these destructive pests (Mathers 2020). The first aphid genome, the pea aphid *Acyrthosiphon pisum*, was sequenced in 2010 (The International Aphid Genomics Consortium 2010). Subsequently, some important pest aphid genomes were sequenced, such as *Aphis gossypii* (Mathers *et al*. 2022), *Myzus persicae* (Mathers *et al*. 2021), and *Sitobion avenae* (Mathers *et al*. 2023). However, these aphids represent only a small fraction of all aphid species, and most published aphid genomes are not assembled at the chromosome level (Mathers *et al*. 2022). This greatly affects large-scale genome structural variation analysis and genome-wide synteny analysis (Chaisson *et al*. 2015; Chakraborty *et al*. 2018). Therefore, high-quality aphid genomic data are needed to understand the diversity, adaptation, and genome evolution of aphids.

The sugarcane aphid, *Melanaphis sacchari*, is one of the major pests on sorghum and sugarcane in many areas of Asia, Africa, Australia, the Far East, and parts of Central, North, and South America (Singh *et al*. 2004; Bowling *et al*. 2016; Pekarcik and Jacobson 2021). *M. sacchari* is difficult to control due to its incredible fertility, rapid population expansion through parthenogenetic reproduction, and its effective dispersal strategy (Brewer *et al*. 2017; Neupane *et al*. 2020). *M. sacchari* feeds on plant phloem nutrients directly harming plant growth and development and also acts as an insect vector causing serious indirect harm to crop production (Hogenhout *et al*. 2008) by transmitting plant viruses, such as sugarcane yellow leaf virus (Ahmad *et al*. 2007) and millet red leaf virus (Blackman and Eastop 1984) in a persistent, circulative, nonpropagative manner (Gray and Gildow 2003), as well as sugarcane mosaic virus (Yang 1986; Setokuchi and Muta 1993) in a nonpersistent manner (Gadhave *et al*. 2020). In addition, *M. sacchari* produces large amounts of honeydew and sooty mold, which results in a serious reduction in yields (Bowling *et al*. 2016). Apart from harming sorghum and sugarcane crops, *M. sacchari* can also damage corn, rice, and so on (Singh *et al*. 2004; Exilien *et al*. 2022). However, a high-quality reference genome is still lacking for *M. sacchari*. In this study, we report a high-quality chromosome-level genome of *M. sacchari* using nanopore long reads and a high-throughput chromosome conformation capture (Hi-C) chromatin interaction map.

## Methods

### Genomic DNA isolation and sequencing

Female *M. sacchari* were collected from the greenhouse at the Hebei Agricultural University, located in Baoding, Hebei Province. The aphids originated from a local wild population native to the region and were named as “Hebei1.” The whole body of insects was collected for short reads, long reads, Hi-C, and mRNA analysis. Genomic DNA was extracted and purified from female *M. sacchari* individuals. For short-read sequencing, we constructed a 150-bp paired-end sequencing library and performed sequencing on the MGISEQ-2000RS platform (MGI Tech Co., Ltd, Shenzhen, China). Trimmomatic (v0.39) (Bolger *et al*. 2014) was used to filter out low-quality bases and remove sequencing adaptors. For long-read sequencing, we quantified the genomic DNA using Qubit (v4.0) (Invitrogen). Then, genomic DNA libraries were prepared using the Ligation Sequencing Kit (Oxford Nanopore Technologies, Oxford, UK: SQK-LSK109) following the manufacturer's protocol and sequenced on an Oxford Nanopore PromethION flow cell.

### Hi-C library preparation and sequencing

To further improve the continuity of the assembled genomes of *M. sacchari*, we generated Hi-C data using chromosome conformation capture experiments. We extracted and purified genomic DNA from whole bodies of female *M. sacchari* individuals. Subsequently, the nuclei were isolated, digested with 100 units of *Dpn*II restriction enzyme, and end-labeled via biotinylation with biotin-14-dATP. The ligated DNA was sheared into 300–600 bp fragments. These fragments underwent end repair, A-tail, and purification steps. Finally, the Hi-C libraries were quantified and sequenced on the DNBSEQ-T7 platform (MGI Tech Co., Ltd).

### RNA library preparation and sequencing

Total mRNA was extracted from whole bodies of female *M. sacchari* individuals using TRIzol reagent (Thermo Fisher Scientific, Waltham, MA, USA) according to the manufacturer's protocol. The RNA-seq libraries were prepared for 150-bp paired-end sequencing on the DNBSEQ-T7 platform (MGI Tech Co., Ltd).

### Genome assembly

Before de novo assembly, we estimated the genome size of *M. sacchari* based on the short reads. Jellyfish (v1.1.10) (Marcais and Kingsford 2011) was employed to calculate the frequency of each of the K-mers (*n* = 17) with the parameters “count -m 17 -s 200000 M –C.” The resulting data were then inputted into GenomeScope (v1.0) (Vurture *et al*. 2017) to estimate the genome size. The preliminary genome assembly was performed using NextDenovo (v2.4.0) (Hu *et al*. 2024) with customized parameters (read cutoff = 10k, seed depth = 45, genome size = 380 M). Subsequently, we used one round of Pilon (v1.24) (Walker *et al*. 2014) polishing with short reads to acquire a corrected assembly. The completeness of the assembly was assessed using Benchmarking Universal Single-Copy Orthologs (BUSCOs) (v4.1.4) (Manni *et al*. 2021) with the Insect_odb10 dataset genes (*n* = 1,367). We used ALLHiC (v0.9.8) (Zhang *et al*. 2019) to obtain a scaffold-level genome based on Hi-C data. The scaffold-level genome was imported into Juicebox (v1.11.08) (Durand *et al*. 2016) and manually corrected to obtain a chromosome-level genome based on Hi-C interaction signals. Finally, 4 chromosomes, namely MSAC_01 (1), MSAC_02 (2), MSAC_03 (3), and MSAC_04 (4 or X), and 132 scaffolds (150 contigs) made up the final primary assembly. During manual correction, scaffolds6–136 with short lengths (0.5–2.1 Mb) could not be clearly located on chromosomes, so we classified them as “unplaced assembly.” Meanwhile, we observed that scaffold5, which is 22.4 Mb and contains 19 contigs, displayed no significant global Hi-C interaction with other regions of the genome except for a weak interaction with part of the chromosome 4. The average coverage of short reads on scaffold5 (∼41-fold coverage) was significantly lower than that of chromosomes 1, 2, 3, and 4 (92–87-fold coverage). Despite annotating 1,321 genes on scaffold5, their expression levels were significantly lower than those of genes on the autosomes and sex chromosomes, with almost no expression. Moreover, the genome and BUSCO gene completeness assessment for scaffold5 was 0%. According to the transposable element (TE) annotation, the proportion of TE was 16.29% in chromosomes 1, 2, 3, and 4 (304.6 Mb), but it was 56.61% in scaffold5 (22.4 Mb). We also found that scaffold5 not only had homologous genes but also exhibited syntenic regions, primarily on the X chromosome. Considering these results, we were unable to place the 22.4-Mb-long scaffold5 on any of the 4 chromosomes of *M. sacchari*. So, we classified it together with 131 other scaffolds as “unplaced assembly.” Subsequent evolutionary and comparative genomic analyses were performed on the chromosome assembly, which includes chromosomes 1, 2, 3, and 4.

### TE and gene annotation

For TE annotation, we constructed a *M. sacchari*-specific repeat database de novo using RepeatModeler (v2.0.1) (Flynn *et al*. 2020) with default parameters. We utilized RepeatMasker (v4.0.9) (http://www.repeatmasker.org) and *M. sacchari*-specific repeat database to identify repeat sequences throughout the *M. sacchari* genome with the parameters: “-e rmblast -div 40 -xsmall -nolow -norna.” Our gene prediction strategy involved a comprehensive approach that combined transcriptome-based, de novo-based, and homology-based methods. For de novo prediction, we employed the AUGUSTUS tool in BRAKER (v2.1.4) (Hoff *et al*. 2016) for gene prediction using BAM files from the RNA-seq alignments as input data. To incorporate homologous evidence into our predictions, we imported protein-coding sequences from *Rhopalosiphum padi* (Mathers *et al*. 2022), *Rhopalosiphum maidis* (Chen *et al*. 2019), *Aphis glycines* (Mathers 2020), and *Aphis fabae* (Mathers *et al*. 2022) into miniprot (v0.11) (Li 2023) using the parameters “-I30 kb –gff” to perform a gene structure analysis based on homologous evidence. In terms of transcriptome-based prediction, raw reads were filtered using Trimmomatic (v0.39). The filtered reads were then mapped to the genome assembly using HISAT2 (v2.2.1) (Kim *et al*. 2019). StringTie (v2.21) (Pertea *et al*. 2015) was used to identify the transcript position in the genome assembly, and transcript sequences were extracted. We further mapped these extracted transcript sequences back to the genomes utilizing PASA (v2.41) (Haas *et al*. 2008). Additionally, TransDecoder v5.50 (https://github.com/TransDecoder/TransDecoder) was employed for generating gene predictions based on PASA-extracted transcripts. Finally, EvidenceModeler (v2.1.0) (Haas *et al*. 2008) [weights for each: Augustus (de novo): 2; TransDecoder (de novo): 3; Miniprot (homology): 8; PASA (transcriptome): 10] was used to integrate gene predictions from all 4 tools. The following parameters were applied during integration: “–segmentSize 10000000 –overlapSize 100000.”

### Phylogenetic tree construction and species divergence time estimation

We estimated a phylogeny of Hemiptera using protein sequences from our new genome assembly of *M. sacchari* and 25 previously reported Hemiptera species, including 6 Aphidini species [*R. padi* (Mathers *et al*. 2022), *R. maidis* (Chen *et al*. 2019)*, A. fabae* (Mathers *et al*. 2022), *A. gossypii* (Mathers *et al*. 2022), *Aphis thalictri* (Mathers *et al*. 2022), and *A. glycines* (Mathers 2020)], 17 Macrosiphini species [*A. pisum* (Mathers *et al*. 2021), *Brachycaudus cardui* (Mathers *et al*. 2022), *Brachycaudus helichrysi* (Mathers *et al*. 2022), *Brachycaudus klugkisti* (Mathers *et al*. 2022), *Brevicoryne brassicae* (Mathers *et al*. 2022), *Diuraphis noxia* (Mathers *et al*. 2022), *Macrosiphum albifrons* (Mathers *et al*. 2022), *Metopolophium dirhodum* (GCF_019925205.1) (https://www.ncbi.nlm.nih.gov/datasets/genome/GCF_019925205.1), *Myzus ligustri* (Mathers *et al*. 2022), *Myzus lythri* (Mathers *et al*. 2022), *Myzus varians* (Mathers *et al*. 2022), *Myzus cerasi (Mathers et al. 2020)*, *M. persicae* (Mathers *et al*. 2021), *Phorodon humuli* (Mathers *et al*. 2022), *Pentalonia nigronervosa* (Mathers *et al*. 2020), *S. avenae* (Mathers *et al*. 2023), and *Sitobion miscanthi* (Mathers *et al*. 2023)], one Eriosomatini species [*Eriosoma lanigerum* (Biello *et al*. 2021)], and one hemipteran outgroup species [*Bemisia tabaci* (GCA_918797505.1)] (https://www.ncbi.nlm.nih.gov/datasets/genome/GCA_918797505.1). In brief, BUSCO (v4.1.4) was used to identify conserved gene orthologs using the insecta_odb10 gene set (*n* = 1,367) in each genome assembly. The identifiers for complete genes present in all species were extracted using python scripts (https://github.com/ypchan/GPA/blob/main/gpa/singel_copy_BUSCO_datasets.py). The protein sequences for each single-copy BUSCO gene from all species were extracted. The sequence alignment of the resulting 832 single-copy BUSCO genes was performed using MAFFT (v7.310) (Katoh and Standley 2013). We extracted conserved sites using Gblocks (v0.91b) (Castresana 2000) with “-b4 = 5 -b5 = h -t = p” parameters after the sequence alignment. The best-fitting model (JTT + F + R6) was determined by the ModelFinder program (Kalyaanamoorthy *et al*. 2017) implemented in IQ-TREE (v2.0.3) (Nguyen *et al*. 2015) based on the Bayesian information criterion and ultrafast bootstrap approximation with 1,000 replicates (-bb 1000). We estimated a divergence time using MCMCTree, a tool within the PAML (v4.9) (Yang 2007) package. Calibration information for fossil nodes was obtained from the TimeTree website (Kumar *et al*. 2022).

### TE divergence distribution

To estimate the relative age of TE and its transposition history in *M. sacchari*, we performed a Kimura distance–based pair divergence analysis of TE superfamilies based on Kimura 2-parameter distances (*K-*values) (Kimura 1980). In brief, we utilized the TE annotation output (alignment files) as input to calculate Kimura distances using calcDivergenceFromAlign.pl and createRepeatLandscape.pl (Perl scripts in the RepeatMasker util directory). Finally, transition and transversion rates were calculated for alignments and transformed into Kimura distances (Kimura 1980) using the following equation: *K* = −1/2 ln (1–2*p*−*q*)−1/4 ln(1−2*q*), where *p* represents the proportion of sites with transitions and *q* represents the proportion of sites with transversions.

### Comparative analysis of orthologous gene families and synteny analysis

To perform a synteny analysis, we employed the following 2 methods: (1) We selected 9 aphid genomes at the chromosome level, encompassing 3 tribes: Aphidini (*R. padi*, *A. fabae*, *A. gossypii*, and *M. sacchari*), Macrosiphini (*A. pisum*, *S. miscanthi*, *B. brassicae*, and *M. persicae*), and Eriosomatini (*E. lanigerum*). OrthoFinder was used to identify strictly single-copy genes for each species. These strictly single-copy genes served as the input for JCVI (v0.7.5) (Tang *et al*. 2024) to plot the chromosomal synteny among species. (2) Syntenic blocks were pairwise identified between species using MCScanX (Wang *et al*. 2012), with a minimum requirement of 5 genes to call a syntenic block (-s 5). MCScanX results were visualized using SynVisio (https://synvisio.github.io/#).

## Results

### Assembly of the *M. sacchari* genome

**Fig. 1.**
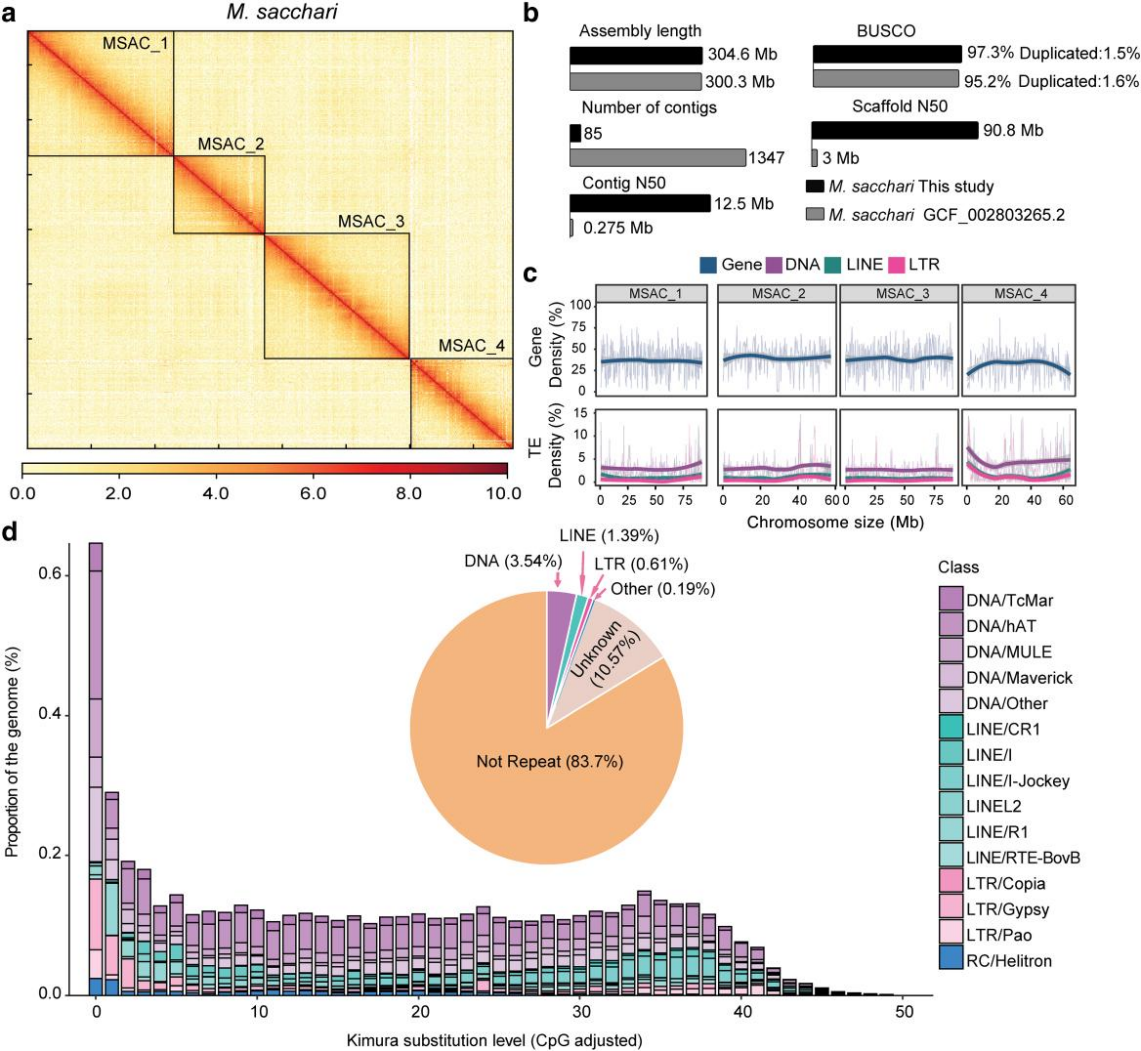
A genome assembly, comparison, and annotation of *M. sacchari*. a) Hi-C matrix of *M. sacchari* genome. A heatmap showing the frequency of Hi-C contacts along our *M. sacchari* genome assembly. The different chromosomes are separated by a black frame. b) A comparison of genome assembly parameters with previously published genomes for *M. sacchari*. c) A density map of gene and TE distribution on chromosomes. Gene and TE density were calculated as the percentage of the length of genes or TEs within a 300-kb window along the chromosomes. d) A repeat landscape of the *M. sacchari* genome. The *x*-axis shows the Kimura 2-parameter distance between repeat copies and their respective consensus sequence, with low score indicating that the repeat copy is more recent. The *y*-axis shows the cumulative percentage of repeats in the genome. TE superfamilies are shown in different colors. A pie chart shows the percentage of TEs of the whole genome. Unknown refers to TEs that were unable to be classified into known superfamilies. A barplot shows the percentage of TEs of known superfamilies. A barplot with unknown repeats is shown in Supplementary Fig. 4.

To assemble a high-quality, chromosome-scale, reference genome of *M. sacchari*, we generated 54.6 million Oxford nanopore ultra-long reads (325 Gb), which is equivalent to ∼915-fold genome coverage (Supplementary Table 1). These reads were assembled using NextDenovo, resulting in an assembly with a contig N50 of 12.5 Mb and a genome size of 355.7 Mb comparable to the estimated genome size of 359.4 Mb (Supplementary Fig. 1). The assembly was polished using paired-end short reads, and contigs were anchored onto chromosome using Hi-C reads (Supplementary Figs. 2 and 3), resulting in a final assembly of 356.1 Mb. The chromosome assembly consists of 4 chromosomes covering 304.6 Mb sequences and containing 85 contigs (Fig. 1a and Supplementary Table 2) (Blackman 1980). In the final assembly, additional 132 contigs or scaffolds, which were 51.5 Mb in size, were classified as “unplaced assembly” (see *Methods* for details). We reported data analysis based on the 304.6 Mb genome sequences composed of 4 chromosomes in the rest of the study. The completeness of the genome was 97.3% (Complete and single-copy or S: 95.8%, Complete and duplicated or D: 1.5%) assessed by BUSCO genes (*n* = 1,367). Compared with a previous genome assembly available at the National Center for Biotechnology Information (NCBI) (GCF_002803265.2), which was highly fragmented, containing numerous gaps, the BUSCO score has increased from 95.2 to 97.3%, and the fragmented BUSCO gene has decreased from 1.3 to 0.4%. Additionally, the number of contigs reduced from 1,347 to 85 and the contig N50 increased from 0.275 to 12.5 Mb (Fig. 1b and Supplementary Table 2). Therefore, the assembled genome of *M. sacchari* was highly contiguous and of high quality.

### Annotation of TEs and protein-coding genes in the *M. sacchari* genome

TEs in the *M. sacchari* genome were annotated using RepeatModeler and RepeatMasker. TEs made up 16.29% of the assembled *M. sacchari* genome (Fig. 1c and d). Most TEs were unknown repetitive elements (10.57%), followed by DNA transposons (3.54%), long interspersed nuclear elements (LINEs) (1.39%), and long terminal repeat (LTR) retrotransposons (0.61%) (Fig. 1d and Supplementary Table 3). Short interspersed nuclear elements (SINEs) were absent in the *M. sacchari* genome, which is consistent with the TE distribution reported in aphid genomes (Baril *et al*. 2023). We observed that chromosome 4 (MSAC_4) exhibited a higher density of TEs compared with other chromosomes, with DNA transposons contributing an average of 5.7% of the sequences and primarily concentrated at the ends of the chromosome (Fig. 1c). In contrast, DNA transposons accounted for ∼2.8–3.1% of other chromosomes in the *M. sacchari* genome. The distributions of sequence divergence for TE superfamilies estimated by Kimura 2-parameter distance indicated that *M. sacchari* experienced a recent transposition peak for both DNA transposons and LTR retrotransposons (Fig. 1d and Supplementary Fig. 4; Kimura 1980; Chalopin *et al*. 2015).

Annotation of protein-coding genes was performed using a combination of homology-based methodology, ab initio predictions, and transcriptome data. A total of 12,530 protein-coding genes were predicted in the *M. sacchari* genome, with 80.2% of them annotated to function using Kyoto Encyclopedia of Genes and Genomes, Gene Ontology, Pfam, or Orthologous Matrix (Supplementary Table 4). The average transcript length was 1,533 bp, which is similar to that of other aphid genomes. The completeness of the protein-coding genes of the *M. sacchari* genome was 95.8% assessed by BUSCO using the insecta_odb10 database (*n* = 1,367), indicating an improvement in single-copy BUSCO genes from 64.1 to 93.3% and a decrease in duplicated BUSCO genes from 31.2 to 2.5% (Supplementary Table 2), indicating the high quality of the *M. sacchari* gene annotation.

### Comparative analysis of genomes among aphids

**Fig. 2.**
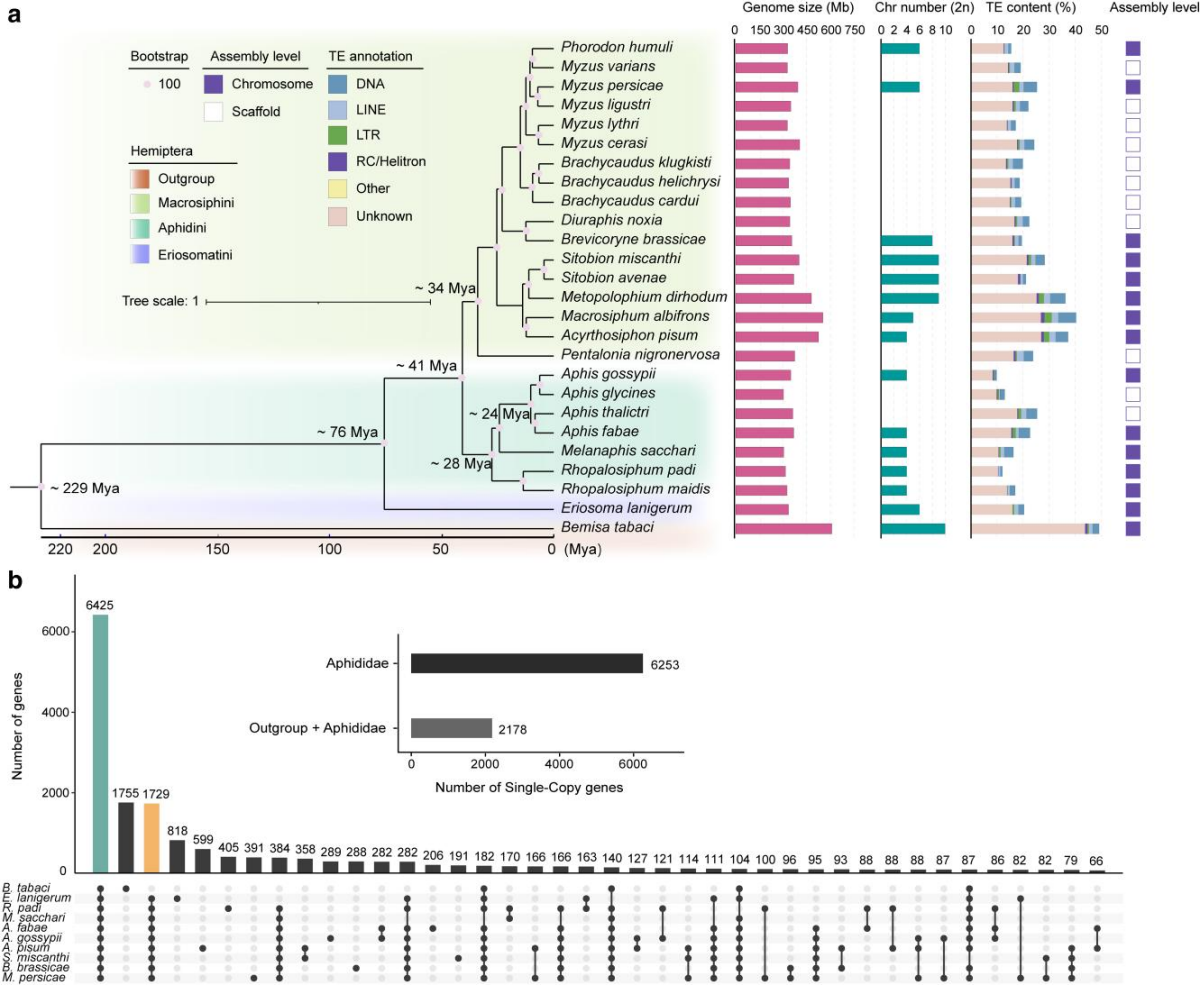
Comparative genomics of the tribes Macrosiphini and Aphidini. a) A phylogenetic tree and characteristic comparison of 25 aphid species. The branch length represents the divergence time, with *B. tabaci* as an outgroup, while each node is supported by a bootstrap value of 100. The tree is annotated with genome size, chromosome number, TE content, and assembly level. b) A comparative analysis of homologous gene families from the 9 aphid species and the *B. tabaci*. The solid black dots in each column represent the set of species corresponding to the number of genes. The first bar on the left indicates the number of genes shared by outgroup and Aphididae. The first and third bars on the left indicate the number of genes shared by the Aphididae. The horizontal bar indicates the number of single-copy genes. Outgroup indicates *B. tabaci*, and Aphididae indicates *M. persica*e, *B. brassicae*, *S. miscanthi*, *A. pisum*, *M. sacchari*, *R. padi*, *A. fabae*, *A. gossypii*, and *E. lanigerum*.

To better understand the evolutionary position of *M. sacchari* within the Aphididae, we incorporated 24 additional Aphididae genomes and used *B. tabaci* as an outgroup to construct the phylogenetic tree (Fig. 2a). Among these 26 species, a total of 832 strictly single-copy genes were identified and used for a phylogenetic analysis. The analysis revealed 3 well-supported clades of Aphididae, namely Macrosiphini, Aphidini, and Eriosomatini (Fig. 2a). The estimated divergence time was ∼76 million years ago (MYA) for these 3 clades, whereas Macrosiphini and Aphidini separated ∼41 (MYA). *M. sacchari* belongs to Aphidini and is diverged from the genus *Aphis* ∼24 MYA. We performed a gene family analysis using 10 representative species with chromosome-level genomes available (Macrosiphini: *M. persica*e, *B. brassicae*, *S. miscanthi*, and *A. pisum*; Aphidini: *M. sacchari*, *R. padi*, *A. fabae*, and *A. gossypii*; Eriosomatini: *E. lanigerum*; and Aleyrodidae: *B. tabaci*). A total of 2,178 single-copy genes and 6,425 genes/gene families were found to be shared in all 10 species; however, Aphididae shared 6,253 single-copy genes and 8,154 genes/gene families (Fig. 2b). This suggests that multiple-copy gene families evolved rapidly in Aphididae. Many gene families were present in a specific lineage or species (Fig. 2b). Additionally, the TE content of the aphid genomes varies from 9.67% in *A. gossypii* to 40.32% in *M. albifrons* (Fig. 2a and Supplementary Table 5). There is a significant positive correlation between the genome size and TE content (*R* = 0.92, *P* = 4.27*e*−11; Supplementary Fig. 5).

### Synteny analysis of aphid genomes

**Fig. 3.**
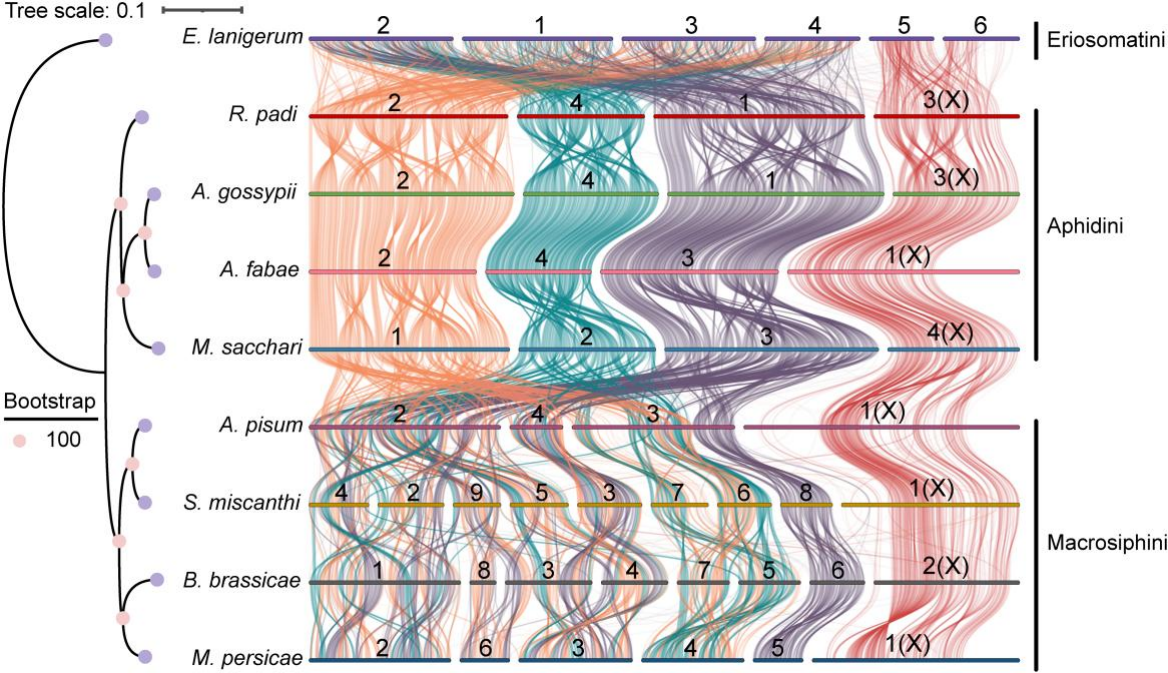
A phylogenetic and chromosomal synteny of single-copy orthologous genes among the aphid tribes Macrosiphini, Aphidini, and Eriosomatini. The left side shows a phylogenetic tree of 9 aphid species. Each line represents a single-copy gene (*n* = 6,253), and the line color is referenced by *M. sacchari*. The number indicates the chromosome numbering of the aphid, with “X” used to denote the sex chromosome.

**Fig. 4.**
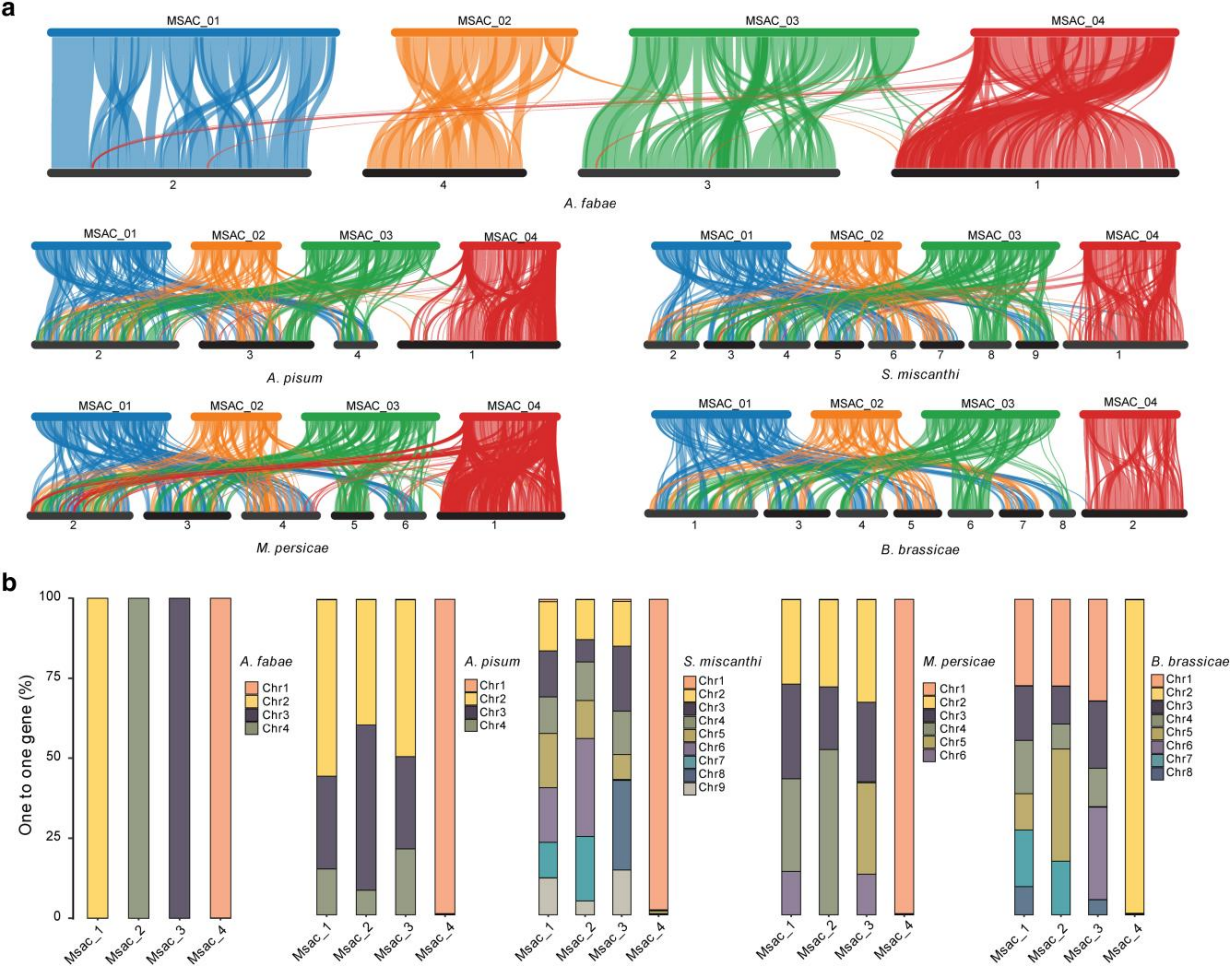
A pairwise synteny analysis of *M. sacchari* and 5 aphids of the tribe Aphidini and Macrosiphini. a) A synteny analysis of *M. sacchari* and other 5 species of aphids (Aphidini: *A. fabae* and Macrosiphini: *A. pisum*, *S. miscanthi*, *B. brassicae*, and *M. persi*cae). The plot shows blocks of syntenic genes. b) A homology of single-copy genes from *M. sacchari* and other 5 species of the tribe Aphidini and Macrosiphini of aphids at the chromosome level. The *x*-axis represents the chromosome on which the *M. sacchari* gene is located. The *y*-axis represents the proportion of genes located on homologous chromosomes that exhibit synteny with another aphid.

To investigate chromosome evolution in *M. sacchari* and other aphids, we performed a synteny analysis between 4 Macrosiphini species (*M. persica*e, *B. brassicae*, *S. miscanthi*, and *A. pisum*), 4 Aphidini species (*R. padi*, *A. fabae*, *A. gossypii*, and *M. sacchari*), and 1 Eriosomatini species (*E. lanigerum*). We first analyzed synteny using single-copy genes and found that *M. sacchari* showed conserved chromosome structure compared to Aphidini species (Fig. 3). However, extensive intra- and inter-chromosomal rearrangements were observed when compared to Eriosomatini and Macrosiphini species, which were consistent with earlier findings reported in a comparative analysis of aphid genomes (Supplementary Fig. 6; Mathers *et al*. 2021, 2023). The analysis also showed that chromosome 4 of *M. sacchari* (MSAC_4) is homologous to X chromosome of *A. pisum*. Aphidini does not exhibit strong rearrangement between X chromosome and autosomal chromosome as in Macrosiphini (Mathers *et al*. 2021, 2023). There is a lack of correspondence between the single-copy genes on chromosome 4 of *M. sacchari* and those located on the arms of the X chromosome within the Macrosiphini, *R. padi*, and *A. fabae*, suggesting that some additional chromosome material appears to have been introduced into the latter species. To further elucidate this phenomenon, we then performed synteny analysis using MCScanX to identify syntenic genome regions based on all protein-coding genes (Fig. 4a). We observed that the protein-coding genes on the arms of X chromosome of *A. pisum*, *S. miscanthi*, *M. persica*e, *B. brassicae*, and *A. fabae* have homologous genes on chromosome 4 of *M. sacchari*, indicating that these genes may arise from gene duplications and accumulate in these regions on X chromosomes of these species. The synteny analysis revealed that homologous genes present on each autosome of *M. sacchari* were identified on the 3 autosomes of *A. pisum*, with over 75% of these genes concentrated on chromosomes 1 and 2 in *A. pisum*. This is also the case when comparing *M. sacchari* to *S. miscanthi*, *B. brassicae*, and *M. persicae* (Fig. 4). Additionally, we observed chromosomal fission and fusion events in *S. miscanthi*, *B. brassicae*, and *M. persicae* (Mathers *et al*. 2023; Li *et al*. 2024). These findings suggest that chromosome fission and fusion events occur less frequently in tribe Aphidini compared to the tribe Macrosiphini, although they diverged within a similar divergence time.

## Discussion

In this study, we assembled a chromosome-level reference genome for the diploid *M. sacchari* with a genome size of 304.6 Mb and a contig N50 of 12.5 Mb. The primary haplotype assembly represents high continuity, accuracy, and integrity compared to the previously released *M. sacchari* genome in the NCBI. Phylogenomic analyses have indicated that *M. sacchari* is a member of the tribe Aphidini and is closely related to the genus Aphis, which includes several agricultural pests, such as *A. gossypii*, *A. fabae*, *A. thalictri*, and *A. glycines*. Therefore, the availability of a high-quality genome of *M. sacchari* will facilitate studying of these important agricultural pests.

In the assembly, we identified a scaffold5, which lacks significant global interaction signals with other chromosomes on the Hi-C interaction map, suggesting that it may be an extra, supernumerary chromosome segment. This is reminiscent of the B chromosomes first discovered in the somatic cells of Hemiptera (Wilson 1907). Scaffold5 exhibits homologous genes and syntenic regions with the other 4 *M. sacchari* chromosomes and the genomes of 8 other aphid species, with these genes and regions predominantly localized to the arms of X chromosome. This observation is analogous to the findings in cichlid fish, where B chromosome sequences were found to be homologous to A chromosome sequences (Ramos *et al*. 2017; Clark *et al*. 2018). In the grasshopper *Eyprepocnemis plorans* and the rodent group Oryzomyini, sex chromosomes appear to be involved in the origin of B chromosomes (Lopez-Leon *et al*. 1994; Ventura *et al*. 2015). The proportion of TEs in scaffold5 is significantly higher than the average levels observed in other scaffolds and chromosomes, yet the types of TEs are fundamentally the same as those found in the A chromosomes. Based on cytological staining patterns, similar phenomena have been reported in animals, plants, and fungi, where B chromosomes exhibit heterochromatic characteristics (Ma *et al*. 2010; Ventura *et al*. 2015). In maize, B chromosome protein-coding gene homologs are widely dispersed across the 10 A chromosomes, without detectable syntenic gene regions of the B chromosome, indicating a high degree of heterogeneity in this region (Blavet *et al*. 2021). However, the absence of clear Hi-C signals presents a challenge in determining whether scaffold5 is indeed a B chromosome. Despite this, we believe that the unique characteristics of scaffold5 could provide valuable material for the study of B chromosomes. In the future, the use of FISH may help us directly observe the specific location of scaffold5 within cells, offering direct evidence for its chromosomal status.

High rates of autosomal chromosome rearrangement have been reported in aphids, such as the formation of *A. pisum* chromosome 3 through a fusion event involving homologues of *M. persicae* chromosomes 4 and 5 (Mathers *et al*. 2021). *A. pisum* chromosome 2 is homologous to 4 small chromosomes in *S. miscanthi* (Mathers *et al*. 2023). In this study, we observed a lower occurrence of inter-chromosome rearrangement events in tribe Aphidini within the same divergence time. A karyotype analysis indicated that most Aphidini species have 4 chromosomes (2*n* = 8), with very few species reaching the highest chromosome number within this tribe of 6 (2*n* = 12) (Blackman 1980). By contrast, the karyotypes of Macrosiphini show a broader range, from 2*n* = 4 to 2*n* = 72. The extensive variability of karyotypes in aphids suggests that different evolutionary forces act on genome evolution in these species, which warrant further investigations. The assembly of the *M. sacchari* chromosome will provide further insights into the intricate evolutionary history of aphid genomes.

## Supplementary Material

jkae223_Supplementary_Data

## Data Availability

The raw sequences of nanopore ultra-long reads (SRR17399617; SRR17399618), whole-genome sequence short reads (SRR17399616), RNA-seq reads (SRR22746183), and Hi-C reads (SRR21203420; SRR21203421) have been deposited in the NCBI SRA (BioProject accession no. PRJNA792680). The genome assembly has been deposited in the NCBI Genome (JBCITD000000000). The assembled genome sequences and gene and TE annotations and the scripts used for analyses are available on Zenodo (https://doi.org/10.5281/zenodo.13283872). All study data are included in the main article and Supplementary Materials. Supplemental material available at G3 online.
