# Supplementary material for: Genome sequence of the sugarcane aphid, Melanaphis sacchari (Hemiptera: Aphididae)
Source: G3 (Bethesda). 2024 Sep 18;14(11):jkae223. doi: 10.1093/g3journal/jkae223 (PMC11540328; doi:10.1093/g3journal/jkae223)
Supplement: jkae223_Supplementary_Data [file jkae223_supplementary_data.zip › Supplemental_Material_Legends_G3-2024-405141.docx]

**Supplementary Figures**

**Figure S1. The k-mer frequency of the *M. sacchari* genome.** The blue columns represent the observed k-mer frequency, while the black fitting line represents the remaining k-mers after removing the portion considered to be sequencing errors (the orange-red fitting line). The yellow fitting lines represent k-mer frequency of non-repeating regions of the genome. The orange-red fitting lines represent to low depth k-mers, which are mostly due to sequencing errors. The vertical dashed black line indicates the integral coverage of the predicted k-mer frequency peaks. The X-axis represents sequencing coverage. The Y-axis represents the frequency of occurrence for 17-bp k-mers in the genome.

**Figure S2.** **Initial genome assembly of *M. sacchari*. A,** Hi-C interaction matrix on the original *M. sacchari* assembly. Blue frames indicate super scaffolds, black frame indicates unplaced scaffolds and green frames show contigs. X and Y axis showing cumulative length in millions of base pairs (Mb). **B,** Depth of sequencing of the initial *M. sacchari* genome. The X-axis indicates the length of the chromosome, and the Y-axis indicates the sequencing depth.

**Figure S3. Collinearity and gene expression analysis of scaffold5. A**, The correspondence of single-copy orthologous genes of *M. sacchari*'s scaffold5 with its own four chromosomes and eight other aphid species**.** **B**, Synteny analysis between scaffold5 and the four chromosomes of *M. sacchari*, as well as eight other aphid species. Blue lines indicate regions of collinearity with the same orientation, while red lines indicate regions with opposite orientation. **C**, Comparison of gene expression levels between the chromosomes and scaffold5 of *M. sacchari*.

**Figure S4.** **Transposable element landscape of the *M. sacchari* genome.** The X-axis shows the Kimura 2-parameter distance of transposable element copies to their respective consensus sequence, with low score indicating young families. The Y-axis shows the cumulative genome percentages of transposable elements. The colored bars represent different transposable element superfamilies.

**Figure S5. Correlation between genome size and TE content in twenty-five aphids.** The X-axis represents genome size, while the Y-axis represents TE content. Pearson correlation coefficients (R) and *P* value were calculated with the R function cor.test. A significance level of *P* = 4.27e-11 indicates a statistically significant correlation.

**Figure S6.** **Chromosomal synteny of single-copy orthologous genes among the aphid tribes Macrosiphini and Eriosomatini.** Each line represents a single-copy gene (n=7,426), and the line color is referenced by *A. pisum*. The number indicates the chromosome numbering of the aphid, with 'X' used to denote the sex chromosome.

**Supplementary Tables**

**Table S1. Raw sequence statistics**

**Table S2. Assembly statistics of the *M. sacchari* genome in this study compared to a previous version (GCF_002803265.2)**

**Table S3. Transposon annotation of the *M. sacchari* genome**

**Table S4. Function annotation of protein-coding genes in *M. sacchari***

**Table S5. Assembly statistics of twenty-five species in Hemiptera**
