# Supplementary figures and images for: Genome sequence of the sugarcane aphid, Melanaphis sacchari (Hemiptera: Aphididae)
Source: G3 (Bethesda). 2024 Sep 18;14(11):jkae223. doi: 10.1093/g3journal/jkae223 (PMC11540328; doi:10.1093/g3journal/jkae223)

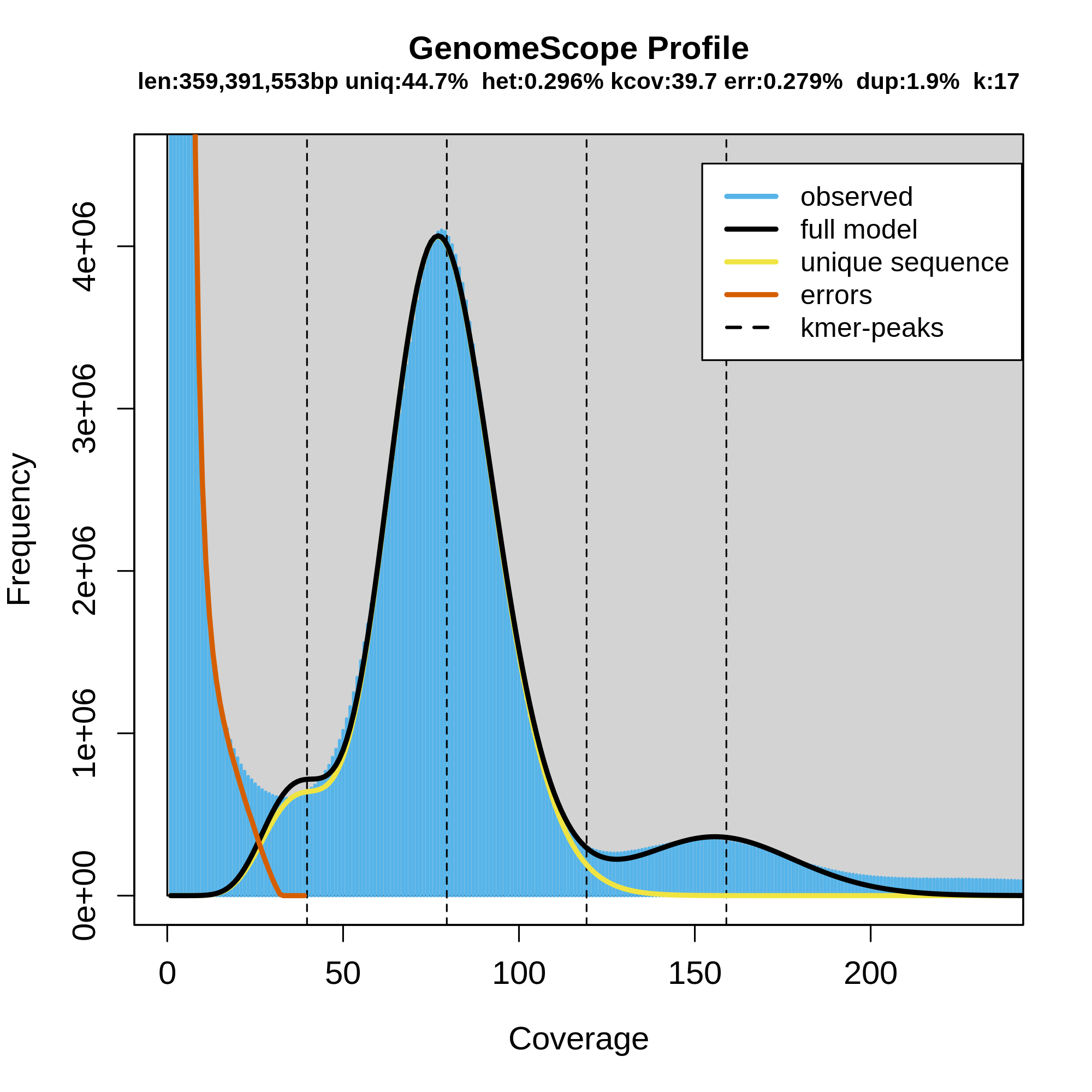

Supplement: jkae223_Supplementary_Data [file jkae223_supplementary_data.zip › Figure_S1_G3-2024-405141.png]

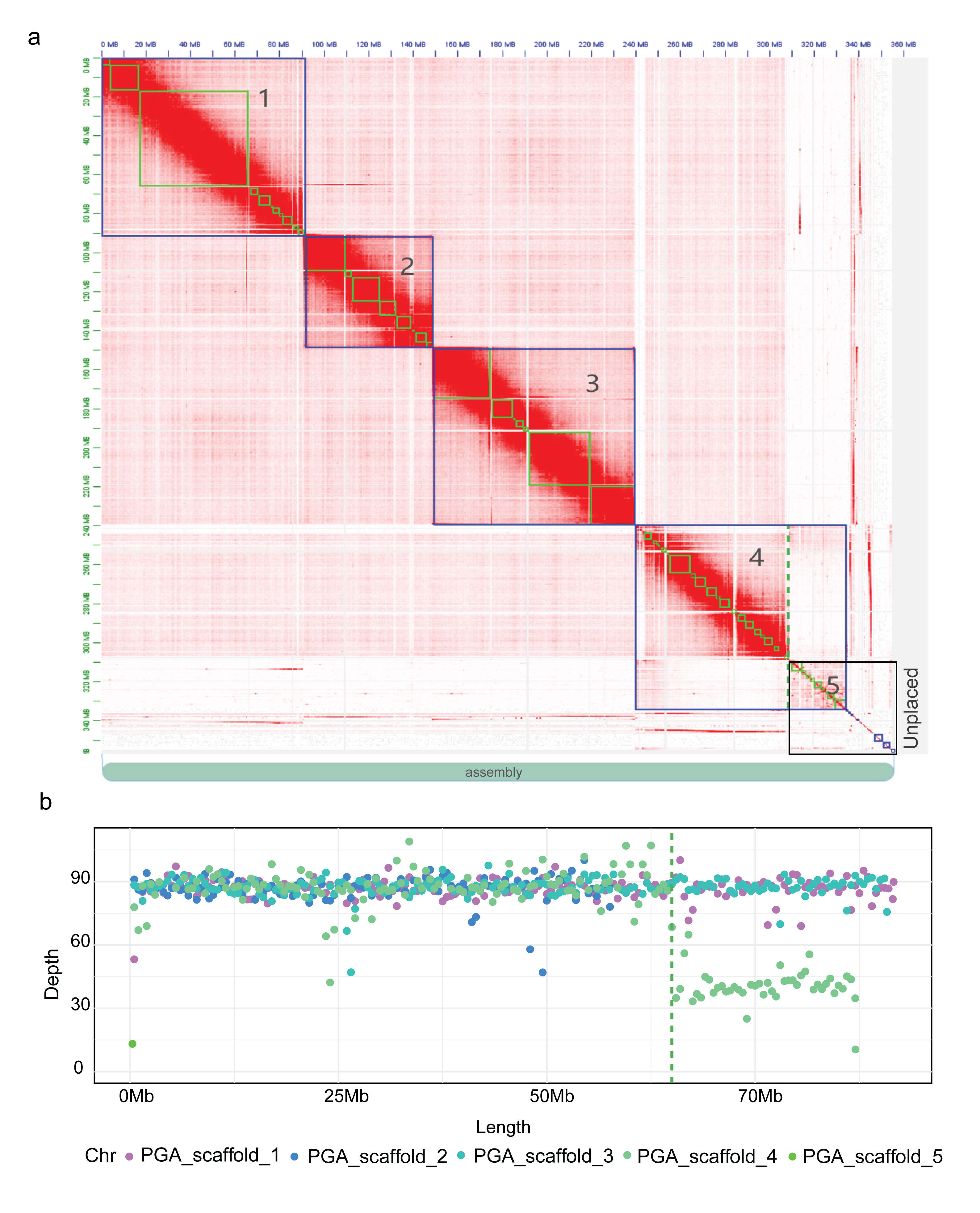

Supplement: jkae223_Supplementary_Data [file jkae223_supplementary_data.zip › Figure_S2_G3-2024-405141.png]

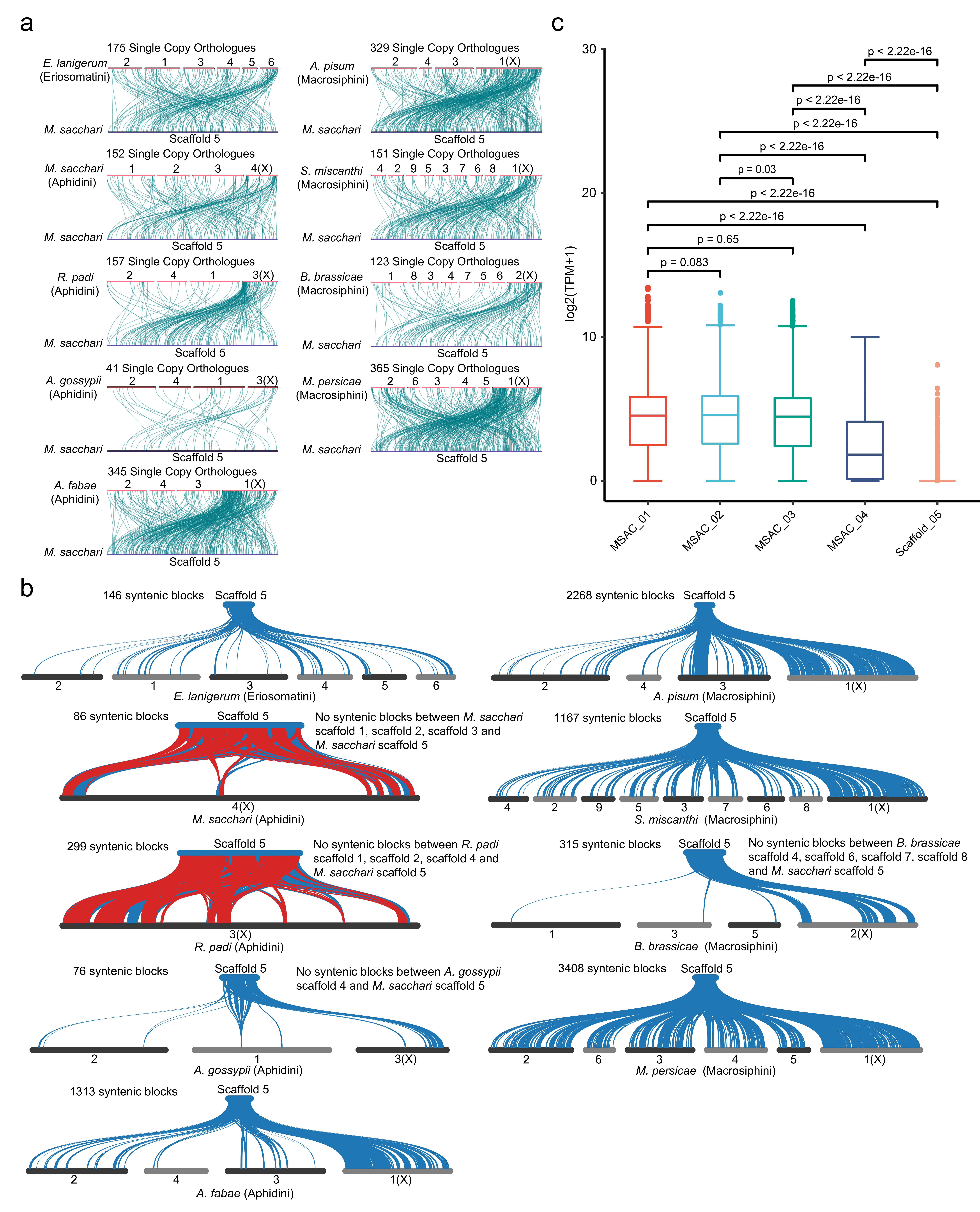

Supplement: jkae223_Supplementary_Data [file jkae223_supplementary_data.zip › Figure_S3_G3-2024-405141.png]

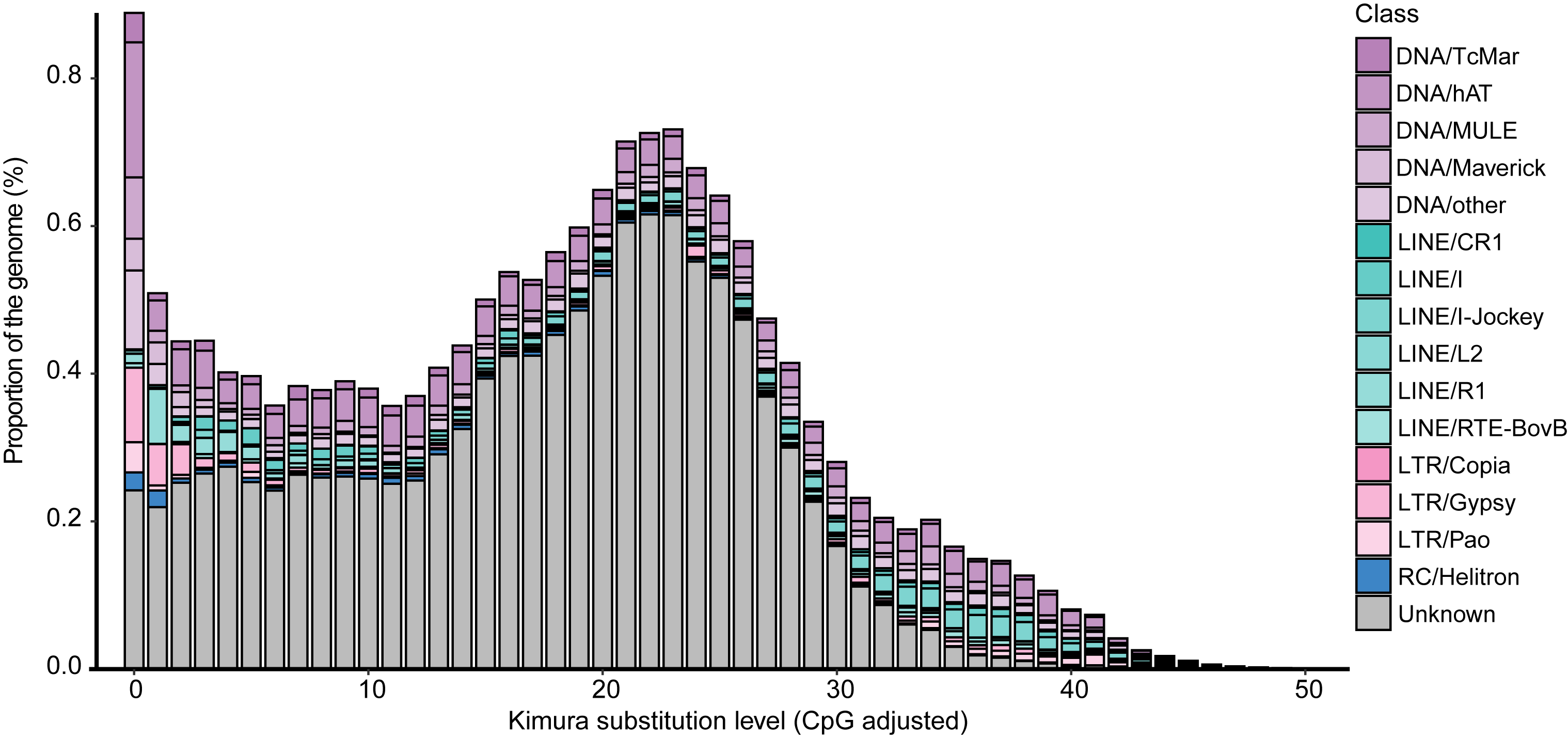

Supplement: jkae223_Supplementary_Data [file jkae223_supplementary_data.zip › Figure_S4_G3-2024-405141.png]

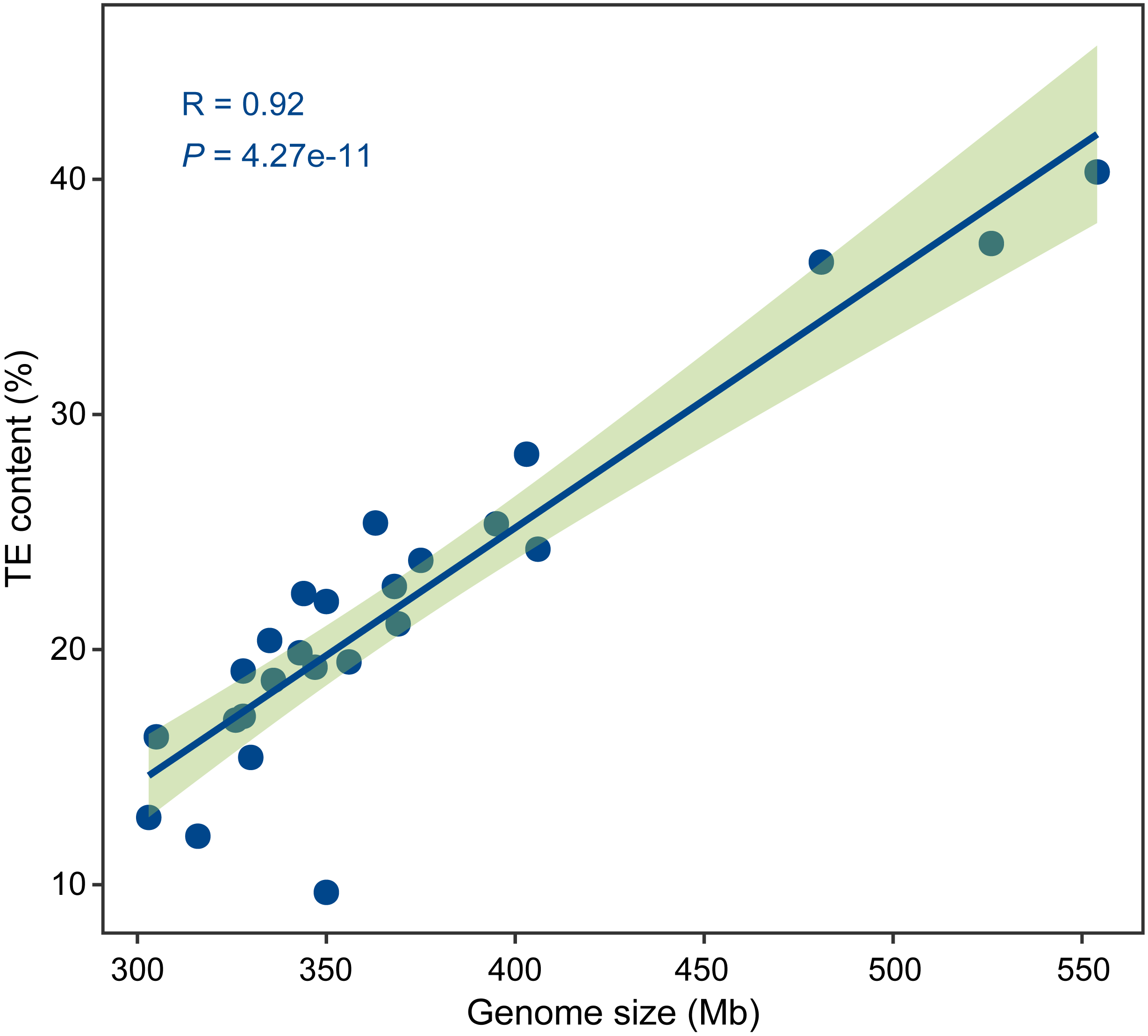

Supplement: jkae223_Supplementary_Data [file jkae223_supplementary_data.zip › Figure_S5_G3-2024-405141.png]

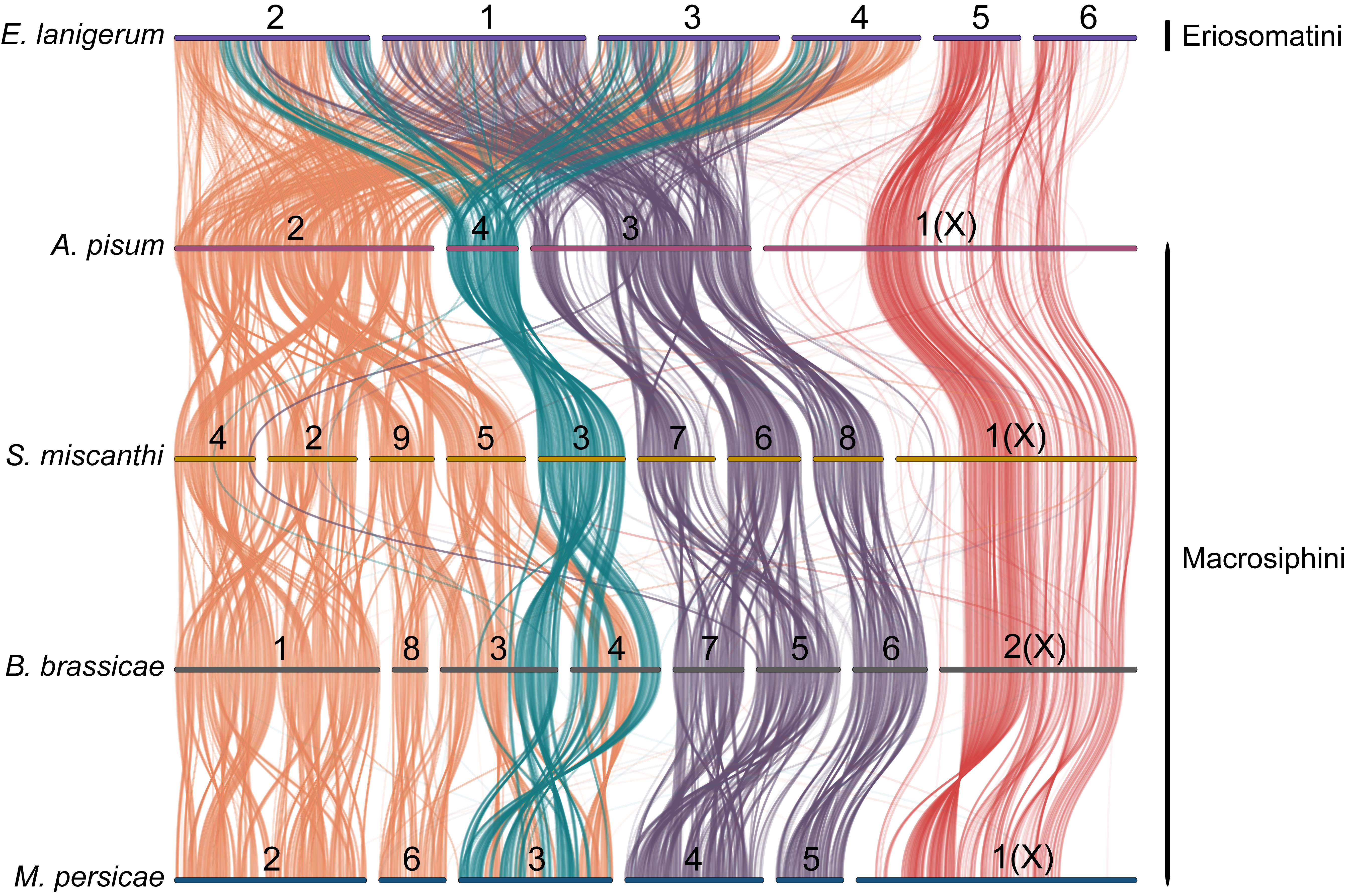

Supplement: jkae223_Supplementary_Data [file jkae223_supplementary_data.zip › Figure_S6_G3-2024-405141.png]
